# Arthroscopic treatment of adhesive capsulitis of the shoulder with minimum follow up of six years

**DOI:** 10.1590/1413-78522015230200613

**Published:** 2015

**Authors:** Marcos Rassi Fernandes

**Affiliations:** 1 Universidade Federal de Goiás, Faculdade de Medicina, Department of Orthopedics and Traumatology, Goiânia, GO, Brazil, 1. Department of Orthopedics and Traumatology, Faculdade de Medicina da Universidade Federal de Goiás, Goiânia, GO, Brazil

**Keywords:** Shoulder pain, Bursitis, Joint capsule release, Arthroscopy, Range of motion, articular

## Abstract

**OBJECTIVE:**

To evaluate the results of the arthroscopic treatment of adhesive capsulitis of the shoulder with six to nine years of follow up.

**METHODS:**

From August 2002 to December 2004, ten patients underwent arthroscopic capsular release for adhesive capsulitis refractory to conservative treatment. An interscalene catheter was used for postoperative analgesia, before the procedure. All were in stage II, with a minimum follow up of six years. The mean age was of 52.9 years old (range, 39 to 66), with female predominance (90%) and six left shoulders. The time between the onset of symptoms and surgery varied from six to 20 months. There were four patients in the primary form (40%) and six in the secondary (60%).

**RESULTS:**

In the preoperative evaluation, the mean active anterior elevation was 92°, 10.5° of external rotation, and internal rotation level L5. Postoperatively, the mean active elevation was 149°, 40° of external rotation and internal level T12, respectively. Thus, the average gains were 57° in forward active elevation, 29.5° in external rotation and six spinous processes, these values being statistically significant (p <0.001). According to the Constant functional score (arc of movements), the value increased from 13.8 (preoperative mean) to 32 points (postoperative mean).

**CONCLUSION:**

Arthroscopic treatment of adhesive capsulitis of the shoulder refractory to conservative treatment allows effective gain of range of motion of this joint*.*

**Level of Evidence IV, Retrospective Study (Case Series).:**

## INTRODUCTION

Adhesive capsulitis (AC), frozen shoulder, stiff shoulder and retractable capsulitis are the terms used to refer to the condition of pain and stiffness of the glenohumeral joint to active and passive movements. This rigidness condition has very diverse etiology, which can be installed primarily in an idiopathic form or secondary to a systemic disease such as diabetes mellitus, hypothyroidism and even subsequently to trauma or surgery in the shoulder articulation.^1^^-^ 3

Several authors have reported that the AC is benign, self-limited and is spontaneously resolved in about two years.^3^ Others, however, show that some patients remain symptomatic with severe movement restrictions, even after several years after the onset of the pathology.^2^

Being a disease that causes great disability, many treatments have been proposed.^3^^,^ 4 Most patients respond adequately to conservative treatment with medication, 3 ^,^ 4 joint infiltrations,^5^ manipulations,^6^ anesthetic block^7^^,^ 8 and/or physiotherapy.^9^ The duration of conservative treatment of AC has been discussed, but the authors have recommended at least six months.^3^

However, some patients do not respond adequately to these treatments, requiring open^10^ or arthroscopic^6^^,^ 11 ^,^ 12 surgical treatment.

Arthroscopy has proven very effective in the treatment of AC for combining minimal tissue trauma and great view of the shoulder joint to the capsular release, besides avoiding the complications of manipulation under anesthesia, such as the proximal humerus fractures.^11^^,^ 12 The hypothesis of the study was that the treatment under arthroscopic view comparing the initial to final results would lead to a significant improvement in shoulder function.

Therefore, we evaluated the results of arthroscopic treatment of refractory shoulder AC, with at least six years of follow-up.

## METHODS

This is a retrospective study (case series) of patients with adhesive capsulitis of the shoulder refractory to conservative treatment, submitted to arthroscopic surgery between August 2002 and December 2004.

In this series were included subjects with: constant and severe pain (zero points in the Constant functional index pain scale); showed no improvement with conservative treatment for at least six months; passive and active shoulder movements limitations (anterior elevation up to 120°, external rotation up to 50° and internal rotation up to L5); situated at stage II of the disease (clinical diagnosis); possession of cognitive conditions to participate; aged between 35 and 70 years old; no significant changes on simple shoulder radiograph; operated by the same surgeon and a minimum follow-up period of six years. Patients with rotator cuff injury and instability according to clinical examination and arthroscopic inspection or those with glenohumeral osteoarthritis, malunion and locked dislocation of the shoulder by imaging studies were excluded from the study.

The sample consisted of 10 patients, aged 39-66 years old (mean 52.9 years old), 90% female and predominantly affected on the left shoulder (60%). Half of the patients presented AC on the dominant hand and mostly in secondary form. (Table 1)

**Table 1.** Clinical and socio demographic data of the studied population.

Case number	Age (years old)	Gender	Side	Dom	Form	Sec	Stage	Seriousness	Time S - O (months	Follow up (years)
1	66	Fem	L	No	Prim		II	Severe	06	9
2	56	Fem	R		Sec	PT	II	Mod	08	8
3	59	Fem	L	No	Sec	PO	II	Mod	09	8
4	39	Fem	R	No	Sec	PT	II	Mod	20	7
5	64	Masc	L		Sec	Diab	II	Mod	09	6
6	47	Fem	L		Prim		II	Mild	07	6
7	45	Fem	R	No	Sec	Hypo	II	Mod	08	6
8	50	Fem	L		Sec	Diab	II	Mod	07	7
9	48	Fem	L	No	Prim		II	Mild	08	6
10	55	Fem	R		Prim		II	Severe	08	6

Fem: feminine; Masc: masculine; R: right; L: left; Dom: dominant limb; Prim: primary; Sec: secondary; PO: post-operation; PT: post-trauma; Diab: diabetes; Hypo: hypothyroidism; Seriousness: seriousness of

Preoperatively, all patients underwent physical therapy with ultrasound, crio and TENS for analgesia and kinesiotherapy for gain in amplitude of movements for a minimum of six months. All patients were administered dexamethasone + cyanocobalamin compounds. Five received lock series of the suprascapular nerve. No hydraulic distension or joint manipulation upon sedation was performed.

As for radiographic evaluation anteroposterior incidences with correction of anteversion of the scapula, axillary profile and scapular profile were performed.

The amplitude of joint mobility was measured, before and after surgery, with the patient supine, compared to the normal side: anterior elevation and 90° elbow flexion external rotation and 0° abduction. Internal rotation was measured by the spinal apophysis reached by the patient's thumb in orthostatism.^13^ The Constant index for clinical and functional evaluation of the operated shoulder was used, considering the variables pain and arc of movement.^14^ In order to classify the disease Zuckerman *et al*.^15^ classification was used. The outcome variable was the amplitude of movement of the shoulder.

Data analysis was performed by Statisticis Package for the Social Sciences (SPSS) version 11.5. The pre- and postoperative values were compared by paired t-parametric test, with risk assumed by the researcher and 5% probability of rejecting the null hypothesis <0.05.

The protocol of this study was approved by the Ethics Committee for Human and Animal Research of Hospital Geral de Goiânia (477- 2009).

### Surgical Technique

An interscalene catheter was inserted for postoperative analgesia before each surgical procedure. Patients under general + brachial plexus block anesthesia were placed in the lateral position with a longitudinal traction device at 20° flexion and abduction of the operated limb and vertical decoaptation of the glenohumeral joint with 5 kg.

The posterior approach of the glenohumeral joint at 2 cm inferior and 2 cm medial of posterolateral edge of the acromion was used. This access was hampered by existing capsular retraction in AC, with due care not to damage the articular cartilage of both the humeral head and the glenoid.

After an inventory of the articular synovium, biceps tendon, humeral head, capsule and rotator cuff, we proceeded to the making of the anterior portal (instrumentation portal), close to the tendon of the long head of the biceps, advancing a 8,25 x 7 mm cannula through the space between rotators from inside-out (in-out).

**Figure 1.**
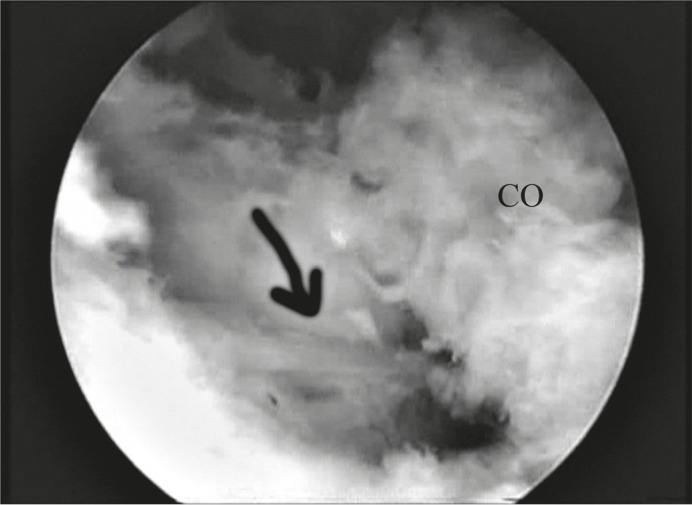
Release of the coracohumeral ligament

Initially a synovectomy was performed with a 4.5 mm full radius blade for opening the rotator interval, from the leading edge of the supraspinatus to the top edge of the subscapularis, also releasing the coracoumeral ligament, which was identified on palpation as a probe. (Figure 1)

**Figure 2.**
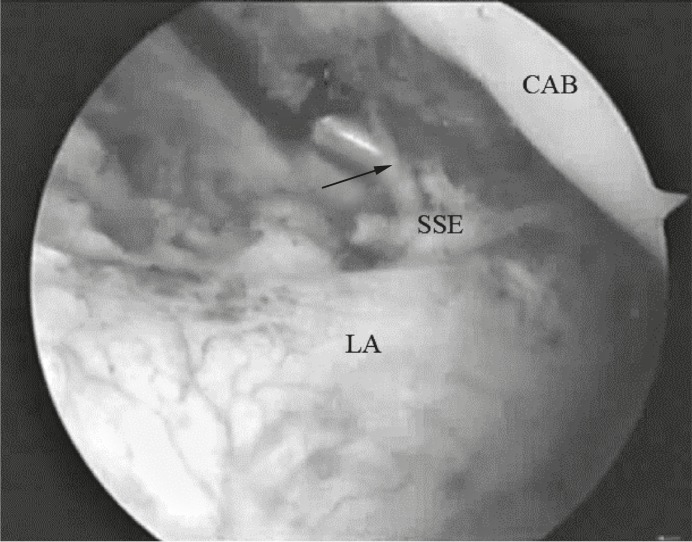
Tenotomy of the subscapular.

Then, using an arthroscopy or radiofrequency electric bistouri, a tenotomy of the middle portion of the subscapularis tendon was performed (Figure 2) lateral to the musculotendinous junction, which was carefully separated from the middle glenohumeral ligament. The opening of the anterior capsule was made by freeing it from the top edge to the bottom edge of the glenoid. Then, the arthroscope was transferred to the anterior cannule, and the electric or radiofrequency scalpel to the posterior portal for the release of the posterior capsule, close to the edge of the glenoid, starting from the back of the biceps to the 8h position.

**Figure 3.**
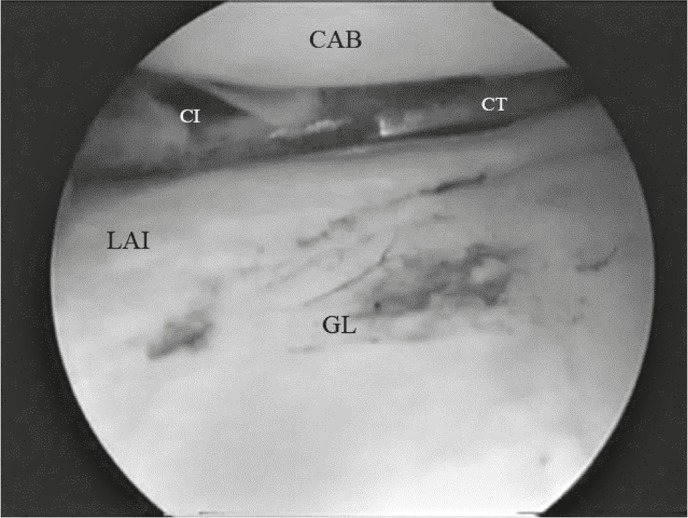
Inferior capsulotomy.

After, the lower capsule was also released (Figure 3) close to the glenoid insertion to complete a circumferential capsulotomy. After the surgical procedure, no manipulation was performed and an increase on the range of motion in all directions was observed. 

Regarding the target population studied, Table 2 shows the additional procedures performed during the arthroscopic procedure, as well as the steps of the surgical technique in surgical cases.

**Table 2.** Arthroscopic and Surgical procedures performed on the studied population

Case number	Anterior C	Superior C	Posterior C	Inferior C	SS Tenotomy	Add Procedure
1	Yes	Yes	No	No	Partial	Acromiplasty
2	Yes	Yes	Yes	Yes	Partial	No
3	Yes	Yes	Yes	Yes	Partial	No
4	Yes	Yes	Yes	Yes	Total	Bursal Synovectomy
5	Yes	Yes	Yes	Yes	Partial	No
6	Yes	Yes	Yes	Yes	Total	No
7	Yes	Yes	Yes	Yes	Total	No
8	Yes	Yes	Yes	Yes	Total	No
9	Yes	Yes	No	No	Total	No
10	Yes	Yes	Yes	Yes	Total	No

C: capsulotomy; SS: subscapular; Add: additional. Source: Medical Files.

There has been physiotherapy in bed twice daily performed by physiotherapist with passive movements and auto passive orientation of the shoulder joint in anterior elevation, external and internal rotation, after infusion of 15-20 mL of 0.5% bupivacaine through the interscalene catheter. Patients remained in hospital for three days with intravenous drug analgesia with 2mL dipyrone each six hours, and 40 mg tenoxicam, each 12 hours. They were released after the improvement of postoperative pain (not assessed by the Constant index), with 100mg oral nimesulide each 12 hours.

In the first four weeks after surgery, patients were submitted to daily physiotherapy sessions, and thereafter each program was individualized, depending on the progress of each patient. Slings were not used and patients were instructed to use the operated limb in daily activities soon after surgery.

## RESULTS

The time between onset of symptoms and surgery ranged from six to 20 months, with an average of nine months.

The mean preoperative active anterior elevation (AE) was 92°; the mean external rotation at 90° elbow flexion and 0° abduction (ER) was 10.5° and the internal rotation (IR) of L5 vertebral level. Postoperatively, the mean AE was 149°; ER was 40° and IR of T12 vertebral level. (Table 3) Therefore, average gains were 57° on AE; 29.5° in the ER and six spinous processes.

**Table 3.** Pre and postoperative values of amplitude of movement of shoulders with adhesive capsulitis operated by arthroscopy.

Case number	AE preop	ER preop	AE postop	ER postop
1	70º	5º	180º	40º
2	90º	30º	120º	40º
3	90º	10º	180º	50º
4	90º	0º	170º	40º
5	100º	10º	120º	20º
6	120º	40º	170º	50º
7	100º	0º	170º	40º
8	90º	10º	120º	50º
9	110º	0º	170º	40º
10	60º	0º	90º	30º
Mean	92º	10,5º	149º	40º

AE: anterior elevation; ER: external rotation; pre-op: preoperative; post-op: postoperative. Source: Medical Files.

Regarding the score on the Constant scale,^14^ with respect to the arc of motion (zero to 40 points), an increase of 13.8 (mean preoperative) to 32 (mean postoperative) was observed. All patients showed decreased in pain scale according to the Constant index in the last assessment (absent or mild).

There were no intraoperative complications, instability or neurological injury postoperatively. Comparing the averages, there were differences in the gain of movements between pre and postoperative (p <0.001). (Table 4)

**Table 4.** Comparison of mean amplitude of movement pre- and postoperative through the parametric t-paired test.

Group	Mean Preop	St. Dev	Mean Postop	St. Dev	t	p
AE	92º	17.51	149º	32.81	5.968	<0.001
ER	10.5º	13.81	40º	9.43	6.743	<0.001

AE: anterior elevation; ER: external rotation; Preop: preoperative; postop: postoperative; St. Dev: Standard deviation; t: test. Source: Medical Files.

## DISCUSSION

Adhesive capsulitis of the shoulder is a common disease, with an uncertain pathogenesis.^16^ Histological characteristics demonstrate a matrix of collagen types I and III, popularized by fibroblasts, suggesting to be modulated by an abnormality in the production of growth factors and citocinas.^17^ This inflammation cascade involves abnormal tissue repair and fibrosis.^18^

Ozaki *et al*.^19^ reported that the contraction of coracohumeral ligament and the rotator interval seems to be the main lesion in CA. The pathological findings of these structures are extremely important when dealing with such patients.

The disease occurs most predominantly between 40 and 60 years old in females on the non-dominant side, without any racial preference.^2^^,^ 3 ^,^ 9 The present study had a mean age of 52.9 years old and 90% of women, which coincides with literature data, although half of the patients presented AC on the dominant side.

Stiff shoulders respond well to non-surgical treatment in 70 to 90% of patients.^4^ Lorbach *et al*.^20^ reported that the use of corticosteroids in both intra-articular injections, as in short-term oral administration improving the range of motion and reducing pain.

Another therapeutic option is blocking the suprascapular nerve, which is an efficient method when compared to placebo and intra-articular injections.^21^ The procedure is appropriate, since this nerve is responsible for 70% of the shoulder capsule sensitivity, which is found retracted and with its volume reduced in AC.^3^^,^ 7 ^,^ 8 However, five of the 10 patients (50%) underwent such a method associated with physiotherapeutic measures without any effectiveness.

Manipulation under anesthesia has been shown effective, but does not allow a controlled release of the pathological tissue with increased risk of humeral fractures.^3^^,^ 5 ^,^ 6 Dodenhoff *et al.* 22 reported that 94% of patients who underwent manipulation were satisfied with its results, however, 12.8% still showed a persistent incapacity. Fox *et al*.^23^ showed that manipulation resulted in sustained improvement in joint function and movements of the shoulder. Due to the risk of complications with this treatment method, it has not been performed in any patient in this series.

Surgical treatment of AC with capsular release should be reserved for patients who do not respond to conservative treatment for at least six months,^3^ which supports this study with the same minimum time from onset of symptoms to the proposed surgery, after unsuccessful conservative measures.

The exploration of the coracohumeral ligament demonstrates that it is the thickest and abnormal part of the capsule in AC.^10^ Being an extra-articular anatomical structure, its arthroscopic release is only possible after opening the rotator interval and exposing the lower lateral surface of the coracoid process. Its section aims to restore external rotation and relief the pain.^19^

This release was performed in all patients of this study and the average gain of external rotation was 29.5°, unlike the study of Beaufils *et al*.^24^ who peformed this procedure in only one out of 26 patients and concluded that capsular release was of little benefit in so called primary AC, with a long recovery time, not leading to any pain relief.

Subacromial fibrosis with hypertrophic synovium was observed in several studies and both debridement as acromioplasty were made for the improvement of results.^25^^,^ 26 Chen *et al.* 27 reported that 86% of the patients underwent subacromial decompression and that this procedure contributed to the relief of shoulder pain. The capsular release was performed in this series, with two additional steps (cases 1 and 4) with substantial pain relief in all cases. Since this study did not aim to associate these two variables, we cannot say that one contributed to the improvement of the other.

In addition to anterior capsulotomy, there is much controversy whether posterior and inferior structures should or not be released. Ogilvie-Harris *et al*.^28^ reported that one should perform the inferior release, but not the posterior. Jerosch^25^ described his technique performing both posterior and inferior release. Chen *et al.,* 27 studying 74 randomized patients, where the first group received only the anterior capsulotomy, while in the second the release was extended to the posterior and inferior capsule, concluded that in six months the function and amplitude of movements of the shoulder were equivalent. Snow *et al*.^11^ also showed no differences when they added the posterior release in the procedure.

This study improved the arc of movement of patients using the posterior and inferior release (except cases 1 and 9), regardless of primary or secondary adhesive capsulitis.

There is also the concern of axillary nerve injury in achieving inferior capsulotomy. As it is closer to the humeral insertion of the capsule, the release should be made near the glenoidal edge.^25^ None of the patients in this series presented neuropraxis of the axillary nerve, the same as Jerosch,^25^ however, Harryman *et al*.^29^ had a praxis case, with spontaneous resolution.

Pearsall *et al*.^26^ and Ogilvie-Harris *et al*.^28^ reported the release of the intra-articular portion of the subscapularis, lateral to the musculotendinous junction, however, most studies show excellent results in the absence of this procedure.^25^^,^ 27 ^,^ 30 This portion represents only 25% of cephalocaudal length of the subscapularis muscle. For this reason and because it is an important restrictor of external rotation, this procedure was added to the presented technique.

Tenotomy made possible not performing any type of joint manipulation in the postoperative period, which ends up being an advantage of the presented technique. It is important to mention that there were no recurrences after surgery. Did the tenotomy contribute to this absence? Since this is not a randomized clinical trial, this question remains unanswered.

It is important to understand whether the subscapularis section undermine the anterior shoulder stability. Pearsall *et al*.^26^ presented 97% of patients with minimal or no signs of instability. Checking the results of this study, there were no cases with anterior instability after arthroscopic surgery, even with partial or total tenotomy.

Berghs *et al*.^31^ presented their results on AC arthroscopic treatment in which the mean anterior elevation improved from 73.7° to 163° (89.3°); the external rotation from 10.6° to 46.8° (36.2°) and internal rotation nine levels. Elhassan *et al.,* 32 analyzing the averages in three directions, obtained an increase of 38°; 24° and six levels, which approximates to the present study that showed an improvement in the average of the 57° on anterior elevation; 29.5° in external rotation and six levels in internal rotation (p <0.001).

Limitations of this study include retrospective design, non-comparative and with a small number of subjects in the sample, since AC is an eminently non-surgical disease, culminating with scattered cases that progress to surgery. This study, however, is important on the aspect of all patients being treated with the same surgical technique, regardless of the etiology of AC, however, their insufficient number in groups do not allow to draw conclusions in this regard. 

## CONCLUSION

Arthroscopic treatment is effective in adhesive capsulitis of the shoulder, resistant to conservative treatment.
